# Fungal deterioration of the bagasse storage from the harvested sugarcane

**DOI:** 10.1186/s13068-021-02004-x

**Published:** 2021-07-02

**Authors:** Na Peng, Ziting Yao, Ziting Wang, Jiangfeng Huang, Muhammad Tahir Khan, Baoshan Chen, Muqing Zhang

**Affiliations:** 1 Guangxi Key Laboratory for Sugarcane Biology & State Key Laboratory for Conservation and Utilization of Subtropical Agro-Bioresources, Guangxi University, Nanning, 530005 China; 2 Sugarcane Biotechnology Group, Nuclear Institute of Agriculture (NIA), Tando Jam, Pakistan

**Keywords:** Sugarcane biodegradation, Post-harvest storing, Fungal community, Yeast, Sucrose losses

## Abstract

**Background:**

Sugarcane is an essential crop for sugar and ethanol production. Immediate processing of sugarcane is necessary after harvested because of rapid sucrose losses and deterioration of stalks. This study was conducted to fill the knowledge gap regarding the exploration of fungal communities in harvested deteriorating sugarcane. Experiments were performed on simulating production at 30 °C and 40 °C after 0, 12, and 60 h of sugarcane harvesting and powder-processing.

**Results:**

Both pH and sucrose content declined significantly within 12 h. Fungal taxa were unraveled using ITS amplicon sequencing. With the increasing temperature, the diversity of the fungal community decreased over time. The fungal community structure significantly changed within 12 h of bagasse storage. Before stored, the dominant genus (species) in bagasse was *Wickerhamomyces *(*W. anomalus*). Following storage, *Kazachstania* (*K. humilis*) and *Saccharomyces* (*S. cerevisiae*) gradually grew, becoming abundant fungi at 30 °C and 40 °C. The bagasse at different temperatures had a similar pattern after storage for the same intervals, indicating that the temperature was the primary cause for the variation of core features. Moreover, most of the top fungal genera were significantly correlated with environmental factors (pH and sucrose of sugarcane, storage time, and temperature). In addition, the impact of dominant fungal species isolated from the deteriorating sugarcane on sucrose content and pH in the stored sugarcane juice was verified.

**Conclusions:**

The study highlighted the importance of timeliness to refine sugar as soon as possible after harvesting the sugarcane. The lessons learned from this research are vital for sugarcane growers and the sugar industry for minimizing post-harvest losses.

**Supplementary Information:**

The online version contains supplementary material available at 10.1186/s13068-021-02004-x.

## Background

Sugarcane is one of the most crucial crops for sugar and bioethanol production [1]. The estimated annual gross sugar output from sugarcane is valued as high as $76 billion [2]. Sugarcane is a perishable commodity and must be processed into sugar quickly after harvested. The post-harvest deterioration of sugarcane is a severe problem for the sugar industry, causing 20–30 percent sucrose losses in cane-producing countries [2–5]. Many factors are associated with the deterioration of the harvested cane, including cane cultivar and its maturity, mechanical or manual harvesting, exposure to microbes, cut-to-crush delay, and storage [2, 5, 6].

The sugar industry prefers pre-harvest sugarcane burning during sugarcane harvesting globally because it reduces transportation costs and makes the harvest process quicker and more accessible [7, 8]. In many countries, the harvested cane is kept in the field for 3–5 days due to the flawed field transport system and 1–3 days in the factory storage under undesirable conditions [2, 5]. Sucrose inversions in the deterioration of harvested cane result from the chemical (acid), enzymic inversion, and microbial activity. In the first 14 h of cane juice deterioration, 93.0% of sucrose losses are caused by microbial, 5.7% by enzymic, and 1.3% by chemical changes (acid degradation) [3]. Microorganism plays a significant role in biodegradation and changes of host chemical characteristics [9, 10]. The high sugar concentration within the mature internodes provides a favorable environment for microbial thriving, which enters the harvested stalk through wounds or cut ends [6]. Also, leaf sheaths and growth cracks provide excellent sites for microbial growth. Over 400 species of bacteria and fungi are associated with sugarcane products [11]. After milling, *Penicillium*, *Lactobacillus*, *Leuconostoc*, and yeast invade the stored sugarcane [2, 12, 13]. These acid-producing microorganisms cause deterioration, decreasing sucrose content, juice purity, and pH, especially under anaerobic conditions such as mud-coated canes and the cane stored in large piles with poor ventilation [5, 13]. The glucose and fructose are converted to organic acids and mannitol by the enzymes secreted by these microorganisms. Besides these external microbes, the endophytic microbial genera viz., *Acetobacter*, *Enterobacter*, *Pseudomonas*, *Aeromonas*, *Vibrio*, *Bacillus*, and *lactic acid* group are also responsible for the deterioration of juice quality during staling [13, 14].

It is crucial to identify the key players in sugarcane deterioration and improve early detection strategies of degradation-causing microbes. The sucrose-related dynamics of fungi in stored sugarcane bagasse remain unexplored. Thereby, the fungal community variations in the stored bagasse were analyzed for assessing the core fungi and specific biodegradation biomarkers using high-throughput sequencing. Moreover, the study also explored the impact of different cane storage durations and temperatures on sugarcane deterioration and dissected these parameters associated with the fungal richness and diversity.

## Results

### Sucrose and pH

**Fig. 1:**
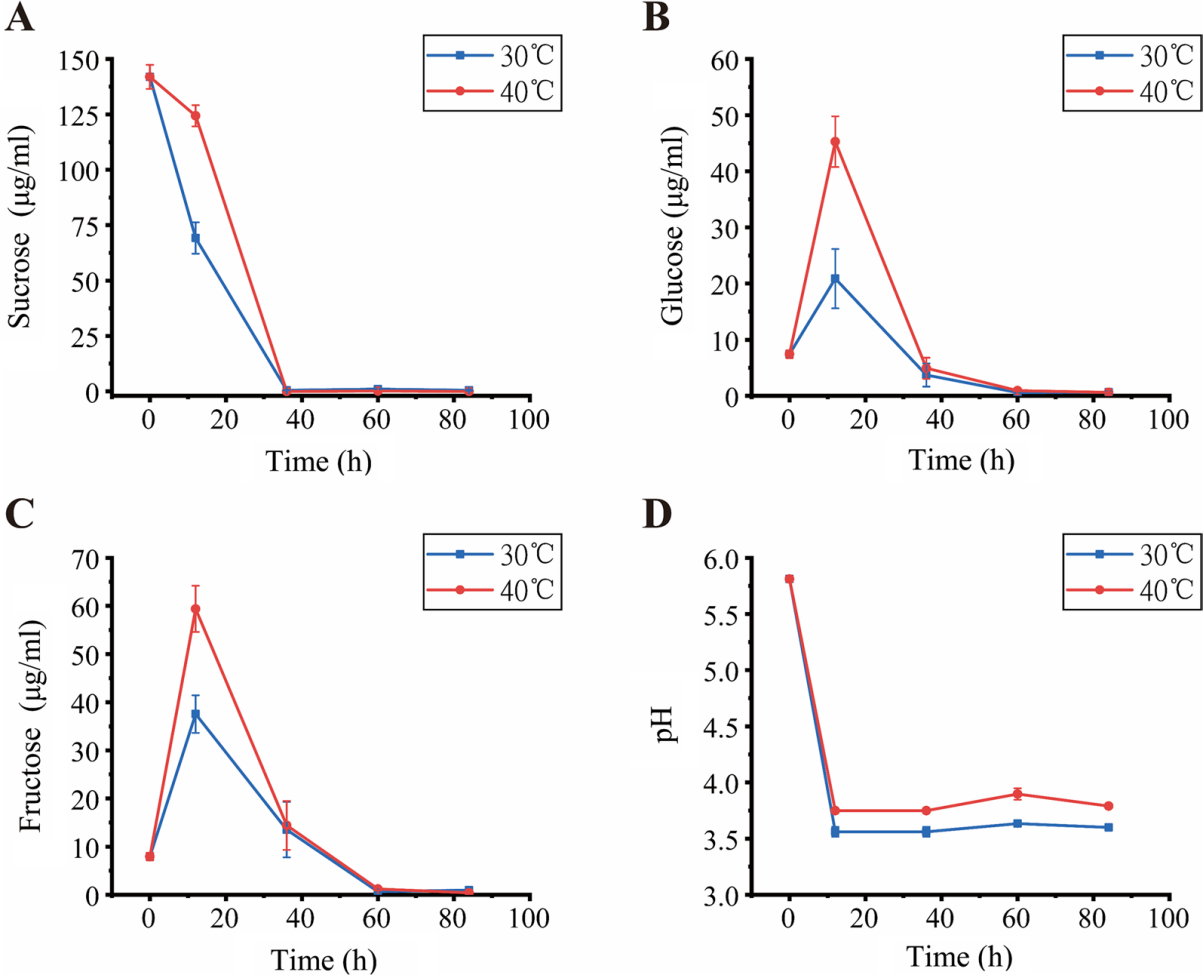
Properties of sugarcane stem samples. Sucrose (**A**), glucose (**B**), fructose (**C**), and pH (**D**) profiles in the stem powder recorded during the storage period. Data were presented as the mean ± standard deviation

In the first 12 h, the steepest decline was observed in the bagasse stored at 30 °C. The sucrose reduced by 53.9% within the first 12 h and then completely decomposed during the initial 36 h for all the treated bagasse. Contrary to sucrose, the glucose and fructose of the bagasse increased in the first 12 h and then fell to zero by 60 h (Fig. 1b, c). However, the glucose and fructose increased in the bagasse stored at 40 °C was higher than those at 30 °C. The pH of all the bagasse reduced significantly in the first 12 h (one-way ANOVA, *p* < 0.001) and then maintained at around 3.6–3.8 (Fig. 1).

### Diversity of fungal communities

**Fig. 2:**
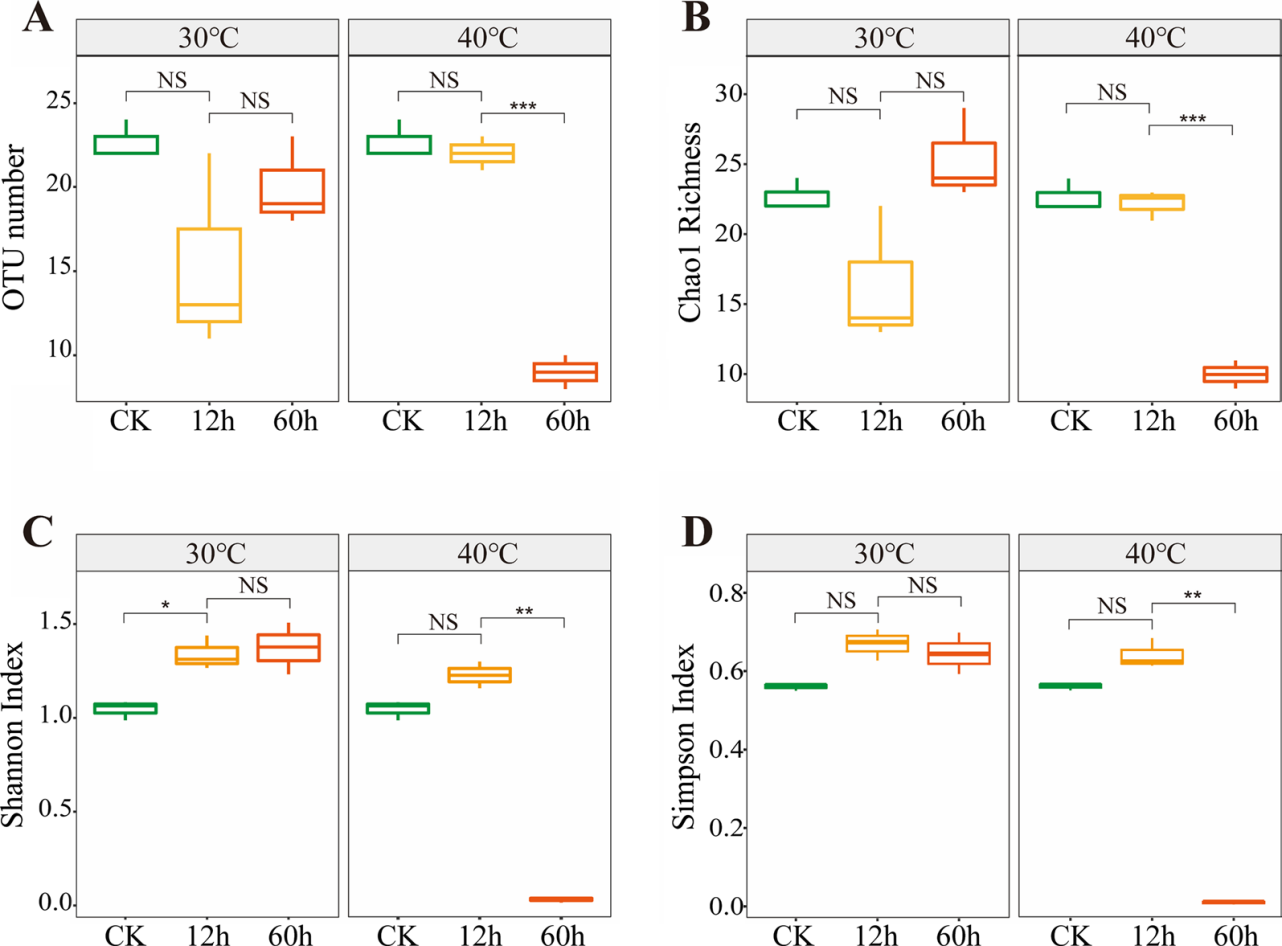
Sequences rarefaction curve and fungal diversity of sugarcane stem samples. Observed OTUs numbers (**A**), Chao richness index (**B**), Shannon diversity index (**C**), and Simpson diversity index (**D**). Each sample was determined in triplicate (*n* = 3). Data were shown as average ± SD. * Indicates significant correlation at p < 0.05, ** indicated significant correlation at *p* < 0.01, and *** indicated significant correlation at *p* < 0.001

**Fig. 3:**
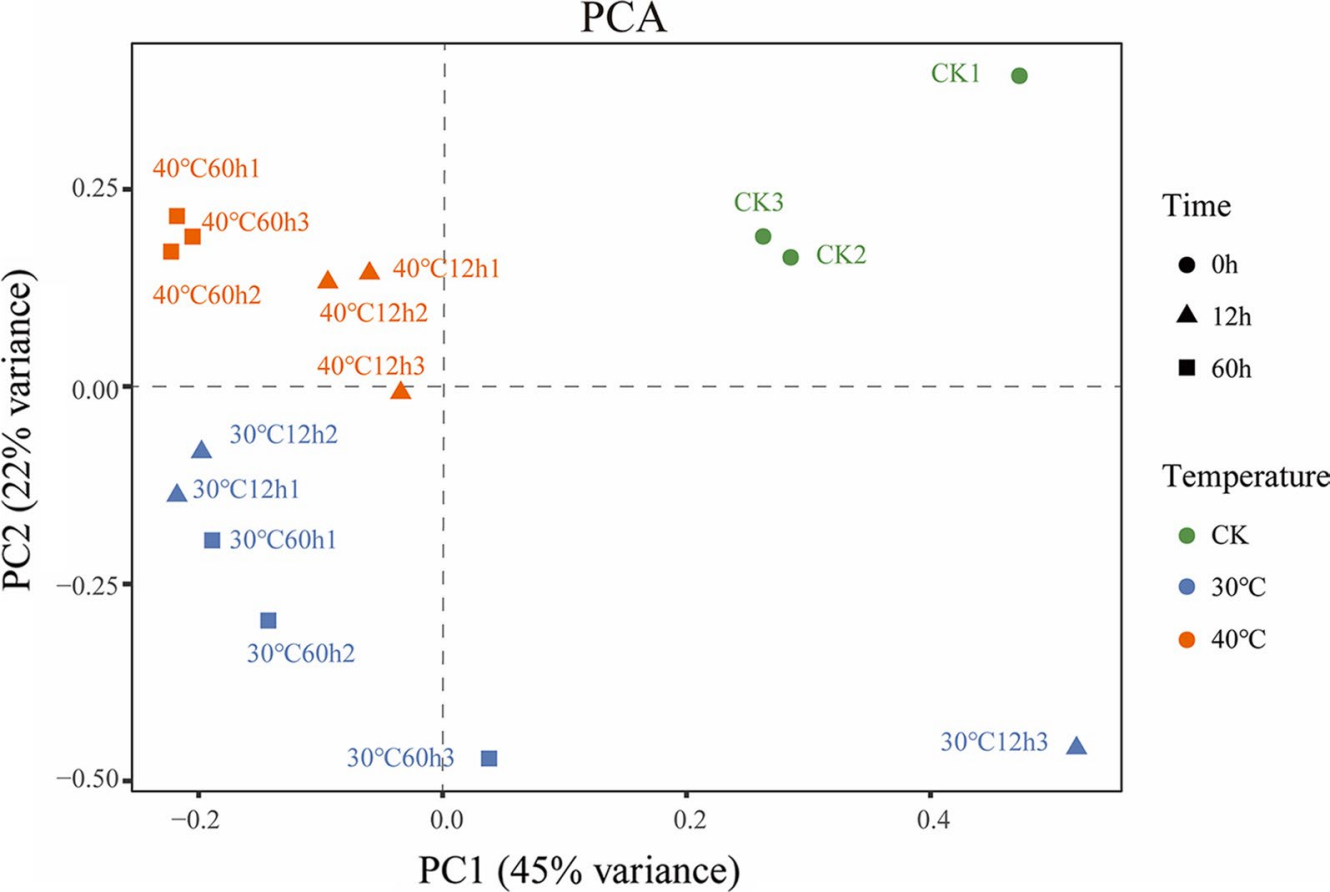
Fungal community structure in sugarcane stem samples. The figure presented principal coordinate analysis (PCoA) of ITS2 sequences diversity in the stored stem at the disparate temperatures used in the study. The different colors indicated the different temperatures of the storage and fungal communities from the sugarcane stem

For determining the fungi prevailing in biodegrading sugarcane, microbial community DNA was extracted and amplified using the ITS2 primer pair (Additional file 1: Fig. S3). Subsequently, the amplicons were sequenced on the Illumina MiSeq platform. A total of 962,640 reads were recovered from all samples after quality filtering (Additional file 1: Tables S1 and S2), representing 31 fungal Operational Taxonomic Units (OTUs) at 97% sequence similarity. The rarefaction curves confirmed that all the samples reached the plateau phase (Additional file 1: Fig. S1). The richness and diversity of fungal OTUs rose in the first 12 h in all the samples. The fungal richness and diversity were significantly lower in the bagasse stored at 40 °C for 60 h than those stored at 30 °C (*p* ≤ 0.05) (Fig. 2). Principal component analysis was used for the OUT data obtained from the 12 treatments and three pre-treatments (CK) after standardization as described in the Methods. The first principal component accounted for 45% of the total variance in the data set, while the second principal component explained 22%. Bagasse stored at 30 °C and 40 °C were quite different from the pretreated bagasse (CK) (Fig. 3). The storage temperature alone explained 34.7% of the microbial community variation (PERMANOVA, *p* = 0.020), while the storage duration explained 73.7% of the variation (PERMANOVA, *p* < 0.001).

Only *Ascomycota* was identified at the phylum level. Four classes were identified at the class level, including *Saccharomycetes*, *Dothideomycetes*, *Sordariomycetes*, and *Eurotiomycetes*, of which *Saccharomycetes* accounted for 99.7%. Five orders were identified, including *Saccharomycetales*, *Pleosporales*, *Hypocreales*, *Chaetothyriales*, and *Eurotiales*, of which *Saccharomycetales* alone accounted for 99.7%. At the family level, ten families were identified, of which *Saccharomycetaceae* accounted for 64.1%, followed by *Phaffomycetaceae* (35.3%). Further, at the genus level, a total of 14 fungal genera were identified. *Saccharomyces*, *Torulaspora*, *Hanseniaspora*, and *Curvularia* were exclusively present in the bagasse stored at 40 °C, while *Kazachstania*, *Zygosaccharomyces*, and *Hanseniaspora* were solely identified in bagasse stored at 30 °C. The dominant genus in CK bagasse was *Wickerhamomyces* (92%).

**Fig. 4:**
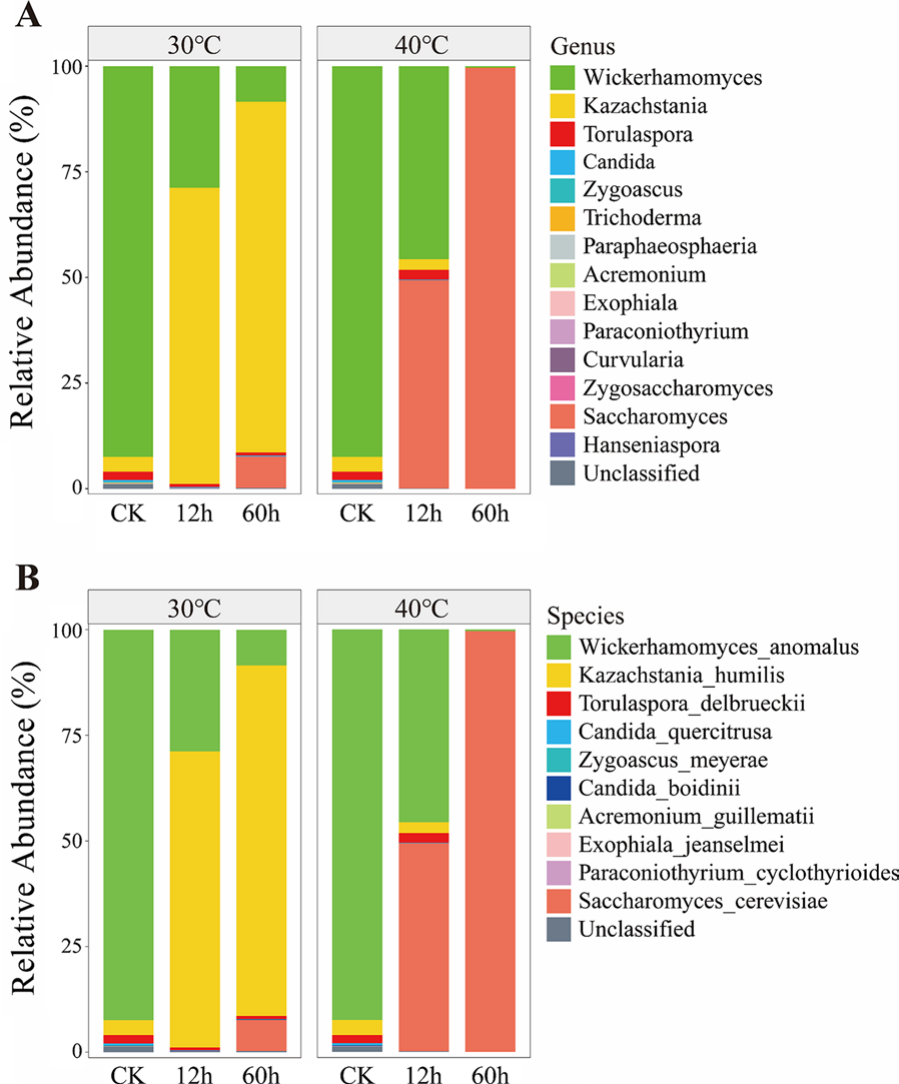
Composition and abundance of microbial communities at the genus (**A**) and species (**B**)

When the bagasses were stored at 30 °C, *Kazachstania* gradually replaced *Wickerhamomyces* as the most abundant fungus (60% at 12 h and 80% at 60 h). However, the bagasses stored at 40 °C for 12 h had two highly abundant genera of *Saccharomyces* (50%) and *Wickerhamomyces* (50%). The relative abundance of *Saccharomyces* increased up to 90% after 60 h in these bagasses (Fig. 4a). At the species level, both *Saccharomyces* (*S. cerevisiae*) or *non-Saccharomyces* yeast (*K. humilis* and *W. anomalous*) were predominant and varied with the increase in temperature and duration of the storage. Before temperature treatment (CK), *W. anomalous* accounted for 92.4% of all tested OTUs. When stored at 30 °C, *K. humilis* was predominant, and its abundance increased from 3.5% (before treatment) to 68.8% after 12 h and 82.9% after 60 h. On the other hand, *W. anomalus* sharply decreased to 8.6% after 60 h of treatment, while *S. cerevisiae* increased to 7.2%. *S. cerevisiae* dominated at 40 °C, accounting for 49.9% after 12 h and 99.6% after 60 h, whereas *W. anomalous* sharply reduced from 44.8% after 12 h to an undetectable level after 60 h. Only 2.4% of *K. humilis* was detected at 40 °C after 12 h of storage (Fig. 4b).

### The primary driver for fungal community composition

**Fig. 5:**
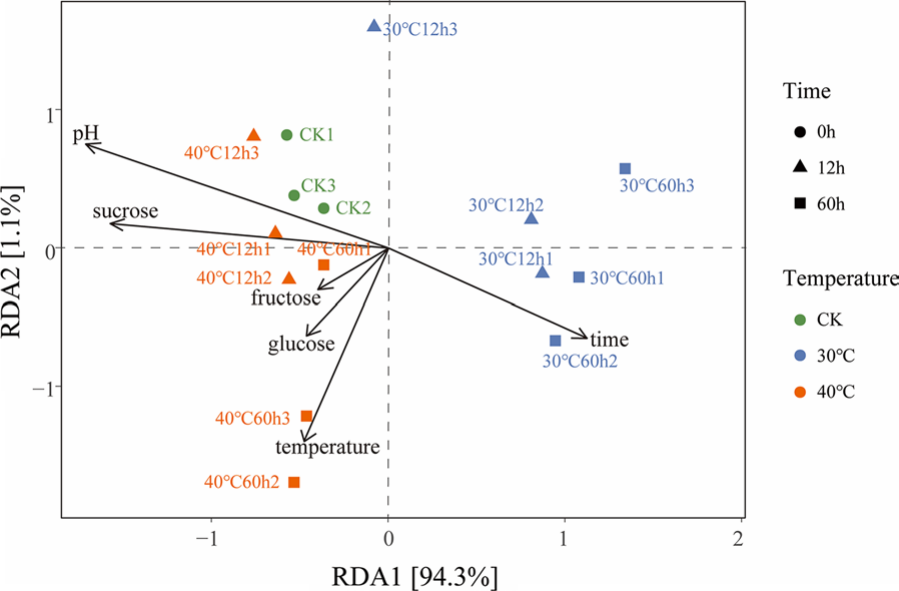
Redundancy analysis (RDA) for the correlation between the fungal community and environmental factors

The redundancy analysis (RDA) was used to determine how the environmental parameters affected the fungal community composition (Fig. 5). Among environmental factors, sucrose content (*F*-ratio = 77.53, *p* = 0.002) and storage time (*F*-ratio = 65.5, *p* = 0.003) are significantly associated with the fungal communities. The storage temperature (*F*-ratio = 30.67) and pH (*F*-ratio = 37.33) also played an essential role in the variation of fungal communities. Hence, RDA showed that the fungal communities varied with the environmental factors.

**Fig. 6:**
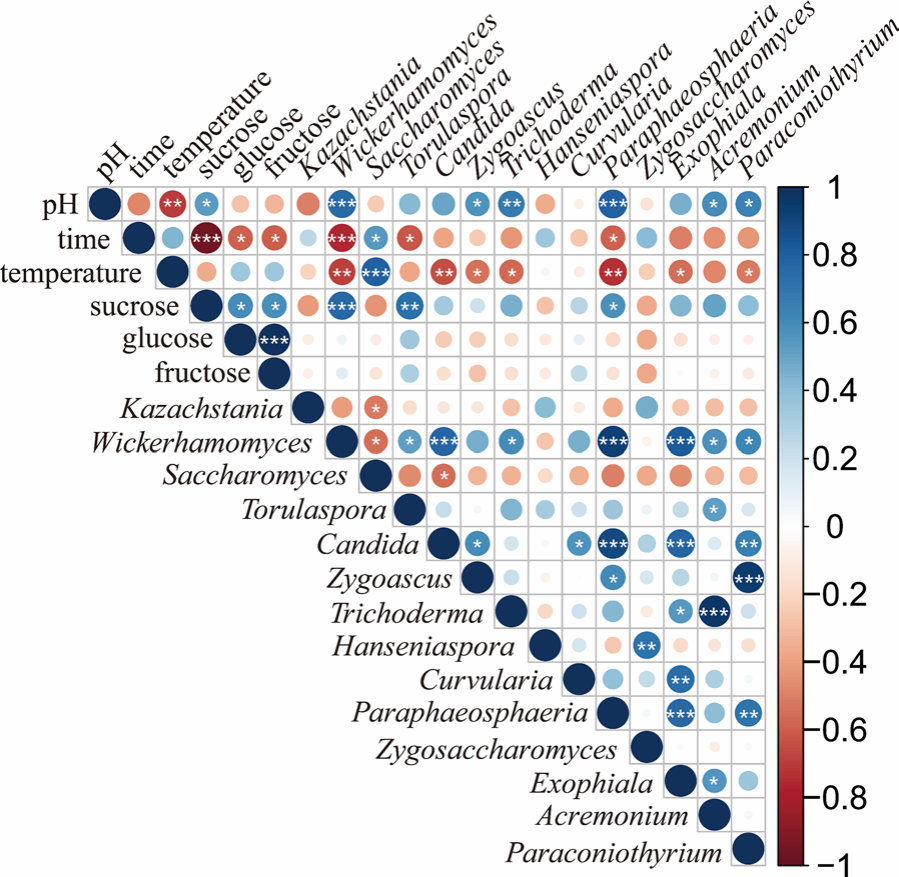
The correlation heatmap of the relationships among the top 14 genera and six selected environmental factors. The minus value of Spearman's correlation index denoted a negative correlation, while a positive value denoted a positive correlation. * Indicated significant correlation at *p* < 0.05, ** indicated significant correlation at *p* < 0.01, and *** indicated significantly correlation at *p* < 0.001

The correlation heatmap of the relationships among the top 14 genera and six selected environmental factors (storage temperature, storage time, sucrose content, fructose content, glucose content, and pH) is presented in Fig. 6. The storage temperature was significantly negatively correlated with pH,* i.e.*, the higher the storage temperature, the lower the pH. This association supported the higher growth of *Saccharomyces* and negatively impacted the richness of any other fungal genera. The storage time had a significant influence on the conversion of sucrose to glucose and fructose. Fructose and glucose content slightly correlated with the fungal abundance, while sucrose significantly affected *Wickerhamomyces* and *Torulaspora*.

### Functional verification of isolated strains

**Fig. 7:**
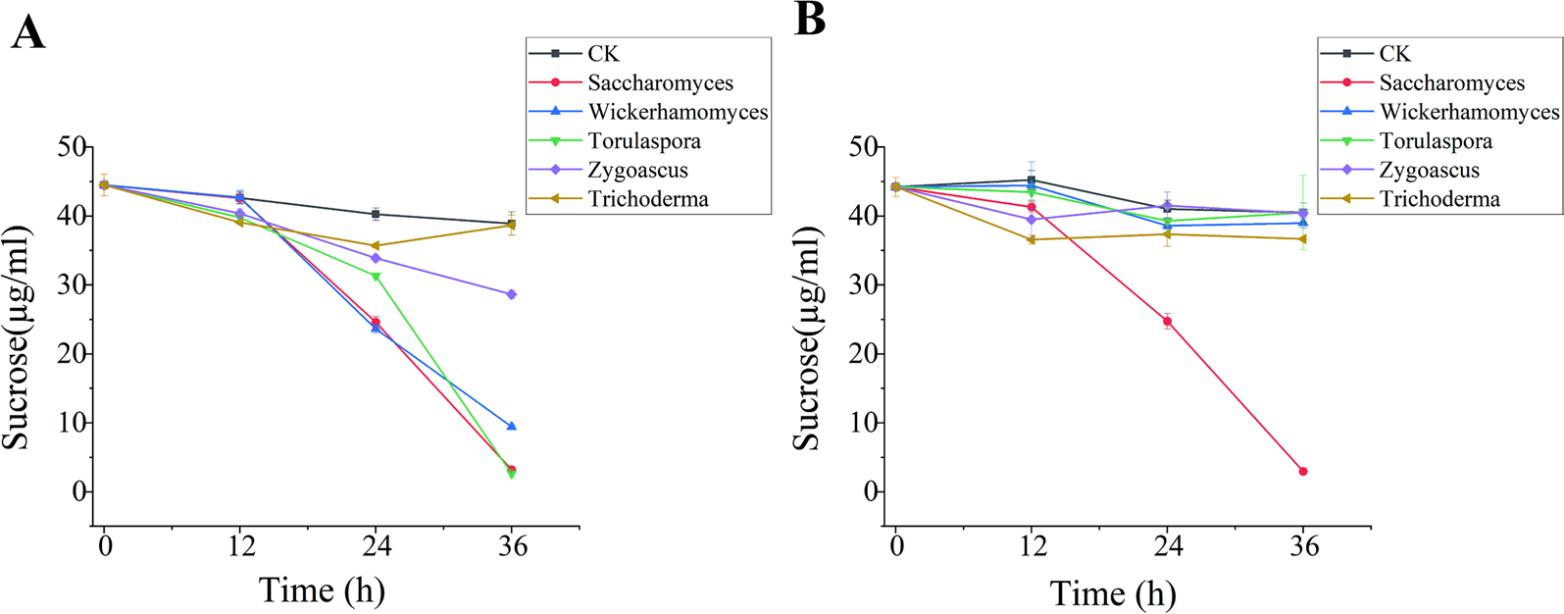
Sucrose profiles in the sucrose medium of the isolated strains grown at 30 °C (**A**) and 40 °C (**B**). Data were presented as the mean ± standard deviation

**Fig. 8:**
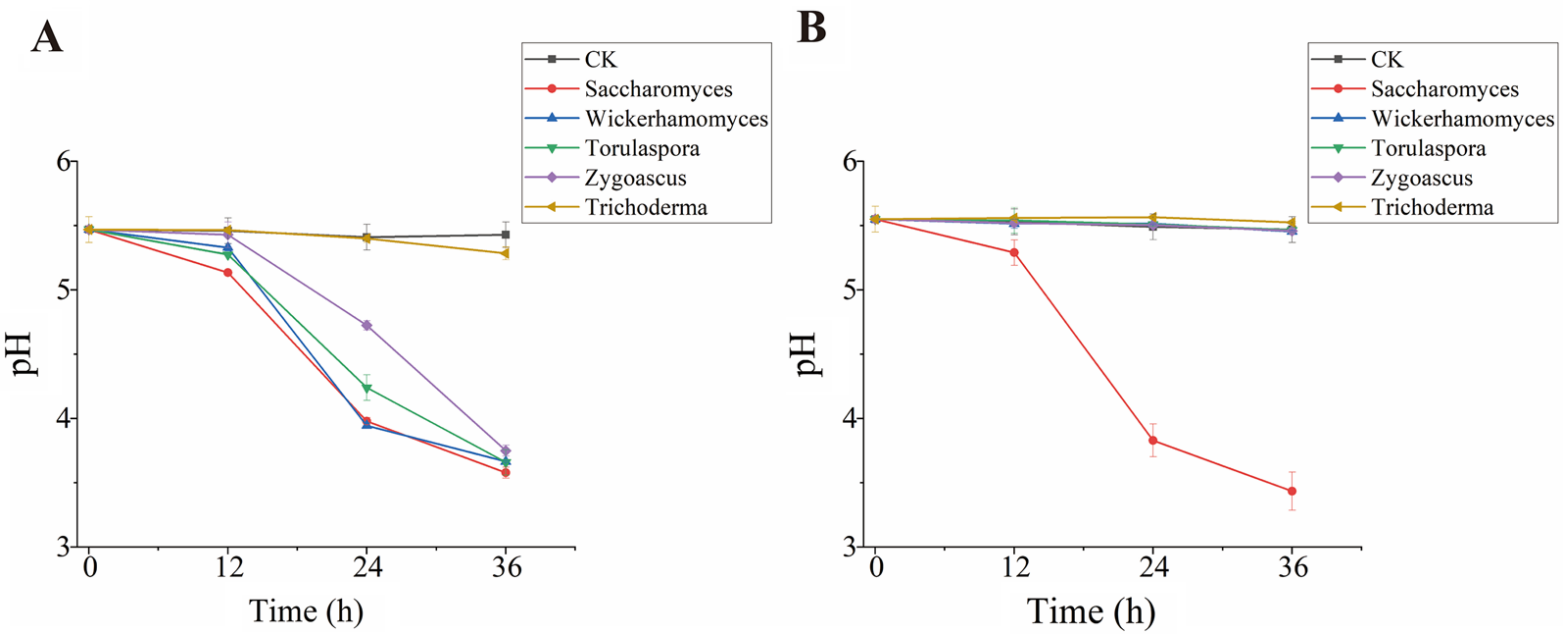
The pH profiles in the sucrose medium of the isolated strains grown at 30 °C (**A**) and 40 °C (**B**). Data were presented as the mean ± standard deviation

Five fungal genera were isolated by plate culture. Further, the utilization of sucrose was assessed by inoculating fungal solutions in the sucrose medium. *Saccharomyces*, *Wickerhamomyces*, and *Torulaspora* had a solid ability to degrade sucrose at 30 °C, consuming sucrose entirely within 36 h (Fig. 7). This phenomenon also yielded different reducing sugar byproducts in different quantifies (Additional file 1: Fig. S2). However, at 40 °C, only *Saccharomyces* maintained the same activity, and the sucrose in its culture medium was completely degraded within 36 h. All other fungi were sensitive to temperature. Consistent with the sucrose contents, the pH dropped in the media culturing the strains. At 30 °C, the pH dropped to 3.6–3.8 after 36 h. The pH of the media having *Saccharomyces* and *Wickerhamomyces* reduced faster (Fig. 8). At 40 °C, only the pH decreased significantly in the medium inoculated with *Saccharomyces*.

## Discussion

The severity of sugarcane deterioration is influenced by many factors, including the length of the harvest-to-crush delay, ambient temperature, and harvesting practices. Pre-harvest burning cane exacerbates post-harvest deterioration rates and requires a short delay of the harvest-to-crush, with a burnt cane left more exposed and reaching higher deterioration levels than green cane [15, 16]. Sugarcane staling is one of the significant issues of the sugar industry, and it causes significant economic losses because of cane and sucrose deterioration during storage and transportation [5, 17]. The post-harvest sugarcane biodegradation necessitates the abating of harvest-to-crush delays. Microbial communities thriving the harvested sugarcane play a crucial role in this deterioration. Moreover, other factors, such as storage time, temperature, and environmental conditions, are essential, too [2, 5, 6]. As per our knowledge, the sucrose-related dynamics of fungi in stored sugarcane remain unexplored. Therefore, this study was conducted to investigate the diversity and abundance of fungal communities in harvested sugarcane. Further, we also examined the impact of different storage temperatures and duration on deterioration processes and fungal abundance.

The pH and sucrose sharply reduced in the first 12 h and then remained stable over 36 h. However, both fructose and glucose increased in the first 12 h, followed by a sharp decrease. The richness and diversity of fungal OTUs declined with the increase in storage temperature and duration. *Wickerhamomyces* (*W. anomalus*), *Kazachstania* (*K. humilis*), and *Saccharomyces* (*S. cerevisiae*) were the most abundant fungal genera (species) in the deterioration of harvested sugarcane. *S. cerevisiae* dominated in the bagasse stored at 40 °C, ranging from 49.9% at 12 h to 99.6% at 60 h. At 30 °C, *K. humilis* enriched from 3.5% in pretreated bagasse to 82.9% at 60 h post-treatment. *W. anomalus* was more prevalent in the pretreated bagasse, decreasing from 92.4 to 8.6% at 30 °C and an undetectable level at 40 °C after 60 h.

*Wickerhamomyces anomalus*, with wide metabolic and physiological diversity, assimilates sucrose, lactose, and starch for its growth [18, 19]. As a classical killer yeast, *W. anomalus* has an antifungal activity to produce protein toxins or low molecular mass glycoproteins lethal to other susceptible yeasts and filamentous fungi (*Aspergillus*, *Botrytis*, *Penicillium*, *Fusarium*) [20–26]. The antifungal properties of *W. anomalus* could be the reason that richness and diversity were very low in our bagasse samples. Only 31 OTUs were assembled from 962,640 reads of our high-throughput sequencing. *W. anomalus* is sensitive to oxygen, whose respiratory growth is favored under aerobic conditions. At the same time, alcoholic fermentation is induced under limited oxygen conditions [19, 27]. *W. anomalus* exhibited growth rates of 0.22 and 0.056 h^−1^ and biomass yields of 0.59 and 0.11 g/g glucose under aerobic and anaerobic conditions, respectively [28]. Therefore, the changes observed in the fungal community structure in our study could be attributed to the fact that since oxygen would have run out in the Ziplock bags during the storage, the fungal communities changed from predominantly respiratory to fermentative. Hence, *Kazachstania* and *Saccharomyces* replaced *Wickerhamomyces* as the most abundant fungi during storage treatment.

*Kazachstania* and *Saccharomyces* are recognized as typical acid-tolerant yeast; they grow well at pH as low as 3.5 [29]. Most species of *Kazachstania* can assimilate sucrose, and several species have similar growth rates and ethanol yields as *Saccharomyces* [30]. The dominance of *S. cerevisiae* over other microbial competitors during fermentation has been traditionally ascribed to its high fermentative power and aptitude to cope with the harsh environmental conditions,* i.e.*, high levels of ethanol and organic acids, low pH, scarce oxygen availability, and depletion of certain nutrients [31–33]. However, *K. humilis* optimally grows at 25–30 °C and does not grow at 37 °C. Therefore, *Kazachstania* was the top genus at 30 °C, while *Saccharomyces* was the dominant genus at 40 °C (Fig. 4). The richness and diversity in the fungal community remained low due to the high sucrose content, acidic pH, and killer toxins.

Pre-harvest burnt cane is more vulnerable to opportunistic microbial infection due to stalk rind splitting and loss of wax integrity, causing seepage of juice and an increased level of exposure [34]. The differences in storage temperature could be the primary cause of the variation of core features. Storage time also played a significant role in influencing community structure and abundance. In agreement with our study, temperature was evaluated as a critical factor in shaping the sugar beet microbial community [35]. Similar findings of rapid deterioration were documented in harvested sugarcane [6]. Although less commonly seen in China due to a low prevalence of mechanical harvesting, billeted or chopped cane is known to deteriorate more rapidly than whole stalk cane due to the larger surface area exposed to infection [36]. Average losses in the untreated billets and whole stalk were 0.735 and 0.502 per day, respectively, indicating that deterioration occurred more rapidly and violently near wounds and cut ends [6]. Cutting the sugarcane stalk in harvesting disrupts the plant physiology. The altered balance among the plant functions leads to many undesirable changes in the composition of the stalk. These adverse effects are worsened by the increased duration of the post-harvest storage period between harvest and mill processing and high ambient temperatures [37]. Due to respiration, an increase in temperature within the heap is likely to have significant consequences for the post-harvest stalk rage and juice quality. Approximately 33% of the energy released in sucrose’s oxidative catabolism is captured in ATP chemical bonds [38]. The remaining energy degenerates as heat that significantly contributes to the warming of the storage piles [37, 39]. This study showed that reducing sucrose percent in the harvested and stored sugarcane increased by the cut-to-crush delay and high atmospheric temperature. Therefore, appropriate measures should be adopted to avoid wounds, cut ends on canes, and complete the crushing in a shorter delay of the harvest-to-crush, keeping ventilation and low temperature as much as possible during transportation, especially for the mechanically chopped cane.

The RDA analysis revealed the pH as another critical environmental factor. We observed that sucrose reduced by 53.9% in the first 12 h. pH was also related to sucrose conversion and declined ahead of sucrose. Similar results have been documented by previous studies as well [40]. A rapid decrease in pH and a rise in titratable acidity during storage increased at higher temperatures [41]. After milling, the acid-producing microorganisms, including *Penicillium*, *Lactobacillus*, *Leuconostoc*, and yeast, increased in the stored sugarcane [2, 12, 13], which lowered the sucrose content, juice purity, and pH, especially under anaerobic conditions [5, 13]. The glucose and fructose were converted to organic acids and mannitol by the enzymes secreted by these microorganisms, which resulted in more deleterious than the simple loss of sucrose [36]. Since pH is very easy to measure, it can be used as a suitable physio-biochemical indicator of post-harvest deterioration of sugarcane.

## Conclusions

Microecology change is considered to be an essential factor of crop deterioration after harvest. This study was conducted to investigate the variation in fungal communities and changes in relevant environmental factors in the post-harvest deterioration of sugarcane. The very low richness and diversity primarily dominated by yeast were observed in the fungal community during the cane storage. *Wickerhamomyces* (*W. anomalus*), *Kazachstania* (*K. humilis*), and *Saccharomyces* (*S. cerevisiae*) were the most abundant fungal genera (species) in the deteriorating cane. The differences in the storage temperature were observed to be the primary cause of variation of core features. Storage time also played a significant role in shaping the fungal community by deciding the relative abundance. The identified top genera in deteriorating sugarcane could be used as a biomarker.

Pre-harvest burnt cane is not permitted in China now, but billeted or chopped cane is becoming more prevalent in mechanical harvesting due to the higher labor cost. The additional effects associated with large-scale stockpiling, mechanical loading, and delivery of cane consignments from the field to the mill were also not considered in this research. For a biomarker to be effective as a post-harvest deterioration indicator, it should become apparent even at low levels of deterioration; it should also be adapted and tested for the type of harvesting system used and the particular type of environmental conditions the cane is exposed. Future work should initially focus on broad-based microbial (fungal and bacterial) investigations under different harvesting and environmental conditions, including a combination of both culture-based and molecular-based analyses, to determine the identities of the microbial populations contributing to the post-harvest deterioration process.

## Methods

### Sugarcane preservation after harvest

A total of 30 healthy sugarcane stalks were sampled from the same field in Fusui, China. Six clean harvested stalks without leaf sheath were surface-sterilized and randomly crushed into bagasse using DM540-CPS. This system rapidly smashed sugarcane with a high-speed rotating blade without pressing or producing cane juice, which the stalks were immediately shredded using DM540 (IRBI Machines & Equipment Ltd, Brazil), blended, and transmitted by CPS (Cane presentation system, Bruker Optik GmbH, Germany) [42]. The cane bagasse of five groups was packed in 50 zip lock bags (500 g each), 40 randomly separated as two groups stored at 30 °C and 40 °C for 4 days. Sampling from each bag was performed at 0 h, 12 h, 36 h, 60 h, and 84 h. A total of 100 g bagasse was blended in cold ddH_2_O using a juicer. The blended solution was filtered through eight sterile bandages and centrifuged at 5,000 rpm for 10 min at 4 °C. The supernatant was stored at − 20 °C, while the pellet, deemed to have epiphytic stalk microbial communities, was stored at − 80 °C. The bagasse filtrates’ pH was determined using a pH meter (Seven Compact, Mettler Toledo). There were three biological replicates in each treatment group.

### Isolation and functional verification of strains

The sugarcane juice was diluted and coated on separation plates. After culturing, different colonies were selected according to colony characteristics for pure culture and molecular identification. Three kinds of culturing plates (PDA, YPD, and WL) were used to obtain isolated fungal genera [43–45]. The isolated strains were inoculated in 1 mL PDW medium, cultured at 30 °C, and 220 rpm for 6 h. Then, 100 µl of the fungal solution was inoculated in a 100 mL sucrose medium, cultured at 30 °C and 40 °C for 0 h, 12 h, 24 h, and 36 h. The culture medium was stored at − 20 °C to determine sucrose, glucose, fructose contents, and pH. The sucrose medium consisted of 20% sucrose, 0.1% glucose, 0.1% fructose, 1% NaCl, and 1% yeast extract by stimulating sugarcane juice composition [46, 47]. The test was carried out in triplicate.

### Soluble carbohydrates quantification by HPAEC-PAD

Soluble carbohydrates (sucrose, glucose, and fructose) were quantified by high-performance anion-exchange chromatography with pulsed amperometric detection (HPAEC–PAD) in a 40 mm × 250 mm CarboPac PA-1 column on an ICS 5000 Dionex System (Thermo Scientific, Waltham, MA, USA). Diluted sample solutions (diluted 1000 times in Mili-Q water) were filtered through a 0.22 μm membrane before injection, and d-lactose monohydrate (Aladdin D1902045, Shanghai, China) was used as an internal standard. The gradient was established by mixing 60% eluant A (H_2_O) with 40% eluant B (500 mmol L^−1^ NaOH) for 5 min, using a flow rate of 1 mL min^−1^ through the column [48, 49]. Each sample was measured twice for technical replicates.

### DNA extraction and sequencing

The community DNA was extracted by the ALFA-SEQ Soil DNA kit (mCHIP, R0911) and quantified by Qubit 3.0 Fluorometer (Life Technologies, Carlsbad, CA, USA) [50]. For amplicon library construction, we used the ITS2 primer pair [ITS21F (5′-CTTGGTCATTTAGAGGAAGTAA-3′) and ITS22R (5′-GCTGCGTTCTTCATCGATGC-3′)]. The PCR mixture contained 5× TransStart FastPfu buffer (4 μL), 2.5 mM dNTPs (2 μL), forward primer (5 μM, 0.8 μL), reverse primer (5 μM, 0.8 μL), TransStart FastPfu DNA Polymerase (0.4 μL), template DNA (10 ng), and ddH_2_O (up to 20 μL). The PCR program was as follows: 95 °C for 3 min, 35 cycles at 95 °C for 30 s, 55 °C for 30 s, and 72 °C for 45 s with a final extension at 72 °C for 10 min. The PCR products were purified using the AxyPrep DNA Gel Extraction Kit (Axygen Biosciences, Union City, CA, USA). The purified amplicons were pooled in equimolar and paired-end sequenced (2 × 300) on an Illumina MiSeq platform (Illumina, San Diego, USA) by Majorbio Bio-Pharm Technology Co. Ltd., Shanghai, China. There were three biological replicates in each treatment group.

### Statistical and bioinformatics analysis

The pH and sugar concentration data were processed using Microsoft Excel and Origin 2016 software and analyzed using SAS statistical software (SAS incorporation, USA).

The raw ITS2 gene sequencing reads were quality-filtered using Trimmomatic, Cutadapt (version 1.9.1) was used to identify and remove primer sequences, and pair-ends were spliced using USEARCH (version 10) [51–53]. Operational Taxonomic Units (OTUs) were clustered at a 97% similarity cutoff. We plotted and calculated a rarefaction curve on the BMKCloud platform. Alpha diversity indices (Chao 1, Shannon, and Simpson index) were determined through Microbiome Analyst, a web-based tool (https://www.microbiomeanalyst.ca/). Principal Components Analysis (PCA) was performed using the “vegan” package in R v. 3.6.3 and plotted on image GP platform (http://www.ehbio.com/ImageGP). The variance explained by parameters was analyzed using a PERMANOVA test in R software (vegan package v. 3.6.3).

The significant differences in relative abundance (average value of three replicates) of fungal genera among treatment groups were determined by one-way variance analysis (*p* ≤ 0.05). Corrplot package in R software was used to analyze the correlation between the top fourteen genera and six selected environmental factors (temperature, storage time, sugar content, and pH). Further, a redundancy analysis (RDA) was performed to investigate the taxa-environment relationships based on OTUs using the “vegan” package in R v. 3.6.3.

## Supplementary Information

**Additional file 1: Table S1.** Sequence characteristics obtained in stored cane stalks. **Table S2.** OTU and Sequence number obtained in stored cane stalks. **Figure S1.** Sequence rarefaction curve. CK: before storage (*n* = 3); s12h30c: stored 12 h in 30 °C (*n* = 3); s12h40c: stored 12 h in 40 °C (n = 3); s60h30c: stored 60 h in 30 °C (n = 3); s60h40c: stored 60 h in 40 °C (*n* = 3). **Figure S2.** The glucose and fructose profiles in sucrose medium of isolated strains grown at 30 °C and 40 °C. (a) Glucose at 30 °C; (b) glucose at 40 °C; (c) fructose at 30 °C; (d) fructose at 40 °C. Data are presented as the mean ± standard deviation. There were three biological replicates in each treatment group. **Figure S3.** PCR amplification for amplicon library construction using ITS21F/ITS22R primer pair. Lane 1: DNA marker; lanes 2–4 (CK): before storage; lanes 5–7 (30 °C 12 h): stored 12 h in 30 °C; lanes 8–10 (40 °C 12 h): stored 12 h in 40 °C; lanes 11–13 (30 °C 60 h): stored 60 h in 30 °C; lanes 14–16 (40 °C 60 h): stored 60 h in 40 °C.

## Data Availability

The datasets supporting the conclusions of this article are included within the article and its Additional files.
