# Supplementary material for: Fungal deterioration of the bagasse storage from the harvested sugarcane
Source: Biotechnol Biofuels. 2021 Jul 2;14:152. doi: 10.1186/s13068-021-02004-x (PMC8254370; doi:10.1186/s13068-021-02004-x)
Supplement: Supplementary file 1 — Additional file 1: Table S1. Sequence characteristics obtained in stored cane stalks. Table S2. OTU and Sequence number obtained in stored cane stalks. Figure S1. Sequence rarefaction curve. CK: before storage (n = 3); s12h30c: stored 12 h in 30 °C (n = 3); s12h40c: stored 12 h in 40 °C (n = 3); s60h30c: stored 60 h in 30 °C (n = 3); s60h40c: stored 60 h in 40 °C (n = 3). Figure S2. The glucose and fructose profiles in sucrose medium of isolated strains grown at 30 °C and 40 °C. (a) Glucose at 30 °C; (b) glucose at 40 °C; (c) fructose at 30 °C; (d) fructose at 40 °C. Data are presented as the mean ± standard deviation. There were three biological replicates in each treatment group. Figure S3. PCR amplification for amplicon library construction using ITS21F/ITS22R primer pair. Lane 1: DNA marker; lanes 2–4 (CK): before storage; lanes 5–7 (30 °C 12 h): stored 12 h in 30 °C; lanes 8–10 (40 °C 12 h): stored 12 h in 40 °C; lanes 11–13 (30 °C 60 h): stored 60 h in 30 °C; lanes 14–16 (40 °C 60 h): stored 60 h in 40 °C. [file 13068_2021_2004_MOESM1_ESM.docx]

Additional file 1

**Table S1 Sequence characteristics obtained in stored cane stalks.**

| **Sample ID** | **PE Reads** | **Raw Tags** | **Clean Tags** | **Effective Tags** | **AvgLen(bp)** | **GC(%)** | **Q20(%)** | **Q30(%)** | **Effective(%)** |
| --- | --- | --- | --- | --- | --- | --- | --- | --- | --- |
| CK1 | 74,223 | 54,585 | 54,584 | 54,584 | 260 | 37.5 | 99.89 | 99.12 | 73.54 |
| CK2 | 71,560 | 55,392 | 55,387 | 55,387 | 261 | 37.49 | 99.88 | 99.12 | 77.4 |
| CK3 | 70,317 | 49,317 | 49,315 | 49,315 | 261 | 37.44 | 99.89 | 99.19 | 70.13 |
| 30℃12h1 | 73,821 | 71,693 | 71,693 | 71,693 | 254 | 44.33 | 99.89 | 99.14 | 97.12 |
| 30℃12h2 | 69,968 | 66,750 | 66,747 | 66,747 | 255 | 44.26 | 99.91 | 99.32 | 95.4 |
| 30℃12h3 | 72,116 | 63,756 | 63,756 | 63,756 | 258 | 39.19 | 99.9 | 99.2 | 88.41 |
| 40℃12h1 | 56,467 | 48,638 | 48,636 | 48,636 | 333 | 37.33 | 99.34 | 96.98 | 86.13 |
| 40℃12h2 | 55,252 | 41,283 | 41,281 | 41,281 | 357 | 37.22 | 99.18 | 96.34 | 74.71 |
| 40℃12h3 | 59,075 | 35,589 | 35,589 | 35,589 | 360 | 37.31 | 99.23 | 96.62 | 60.24 |
| 30℃60h1 | 74,617 | 71,293 | 71,291 | 71,291 | 268 | 43.82 | 99.76 | 98.63 | 95.54 |
| 30℃60h2 | 73,684 | 68,561 | 68,559 | 68,559 | 280 | 42.81 | 99.68 | 98.37 | 93.04 |
| 30℃60h3 | 72,162 | 64,696 | 64,695 | 64,695 | 257 | 43.69 | 99.87 | 99.05 | 89.65 |
| 40℃60h1 | 47,140 | 46,581 | 46,581 | 46,581 | 435 | 37.14 | 98.79 | 94.87 | 98.81 |
| 40℃60h2 | 44,949 | 44,415 | 44,411 | 44,411 | 435 | 37.11 | 98.76 | 94.64 | 98.8 |
| 40℃60h3 | 47,289 | 45,785 | 45,785 | 45,785 | 436 | 37.14 | 98.93 | 95.2 | 96.82 |

CK: before storage (n= 3); 30℃12h: stored 12h in 30℃ (n =3); 40℃12h: stored 12h in 40℃ (n =3); 30℃60h: stored 60h in 30℃ (n =3); 40℃60h: stored 60h in 40℃ (n =3).

**Table S2 OUT and Sequence number obtained in stored cane stalks.**

| Sample ID | OTU number | Sequence number |
| --- | --- | --- |
| CK1 | 28 | 54256 |
| CK2 | 25 | 55148 |
| CK3 | 26 | 48968 |
| 30℃12h1 | 14 | 71681 |
| 30℃12h2 | 11 | 66719 |
| 30℃12h3 | 25 | 63541 |
| 40℃12h1 | 25 | 48539 |
| 40℃12h2 | 24 | 41159 |
| 40℃12h3 | 25 | 35463 |
| 30℃60h1 | 19 | 71257 |
| 30℃60h2 | 20 | 68466 |
| 30℃60h3 | 26 | 64550 |
| 40℃60h1 | 9 | 46526 |
| 40℃60h2 | 10 | 44344 |
| 40℃60h3 | 8 | 45761 |
| Total | 28 | 826378 |

CK: before storage (n= 3); 30℃12h: stored 12h in 30℃ (n =3); 40℃12h: stored 12h in 40℃ (n =3); 30℃60h: stored 60h in 30℃ (n =3); 40℃60h: stored 60h in 40℃ (n =3).

**Figure legends:**


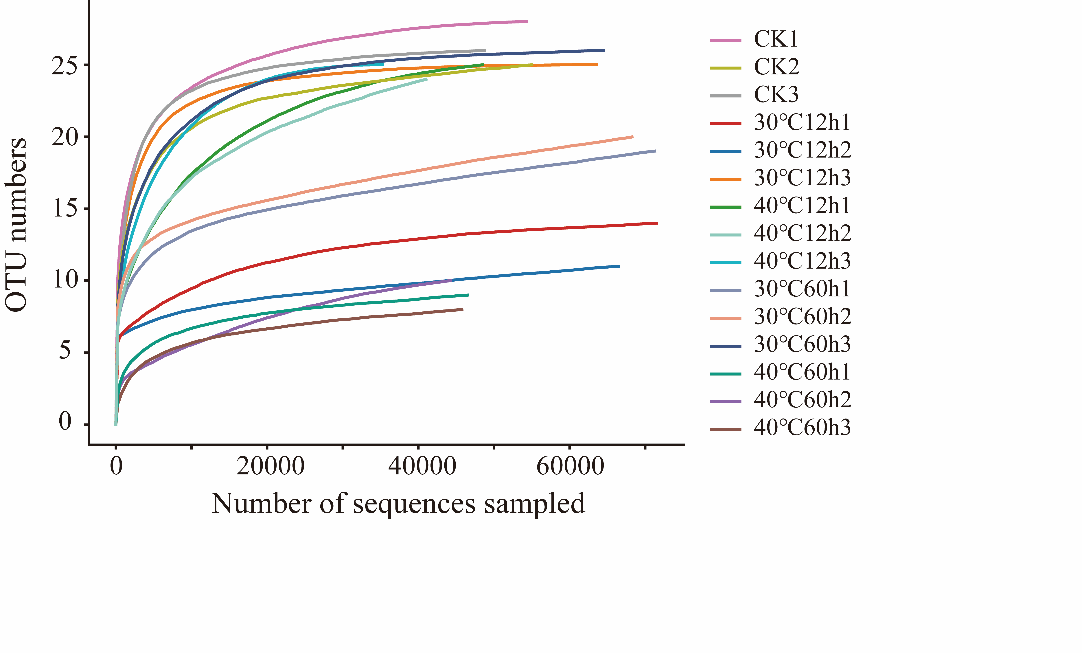


**Fig S1. Sequence rarefaction curve**

CK: before storage (n= 3); 30℃12h: stored 12h in 30℃ (n =3); 40℃12h: stored 12h in 40℃ (n =3); 30℃60h: stored 60h in 30℃ (n =3); 40℃60h: stored 60h in 40℃ (n =3).


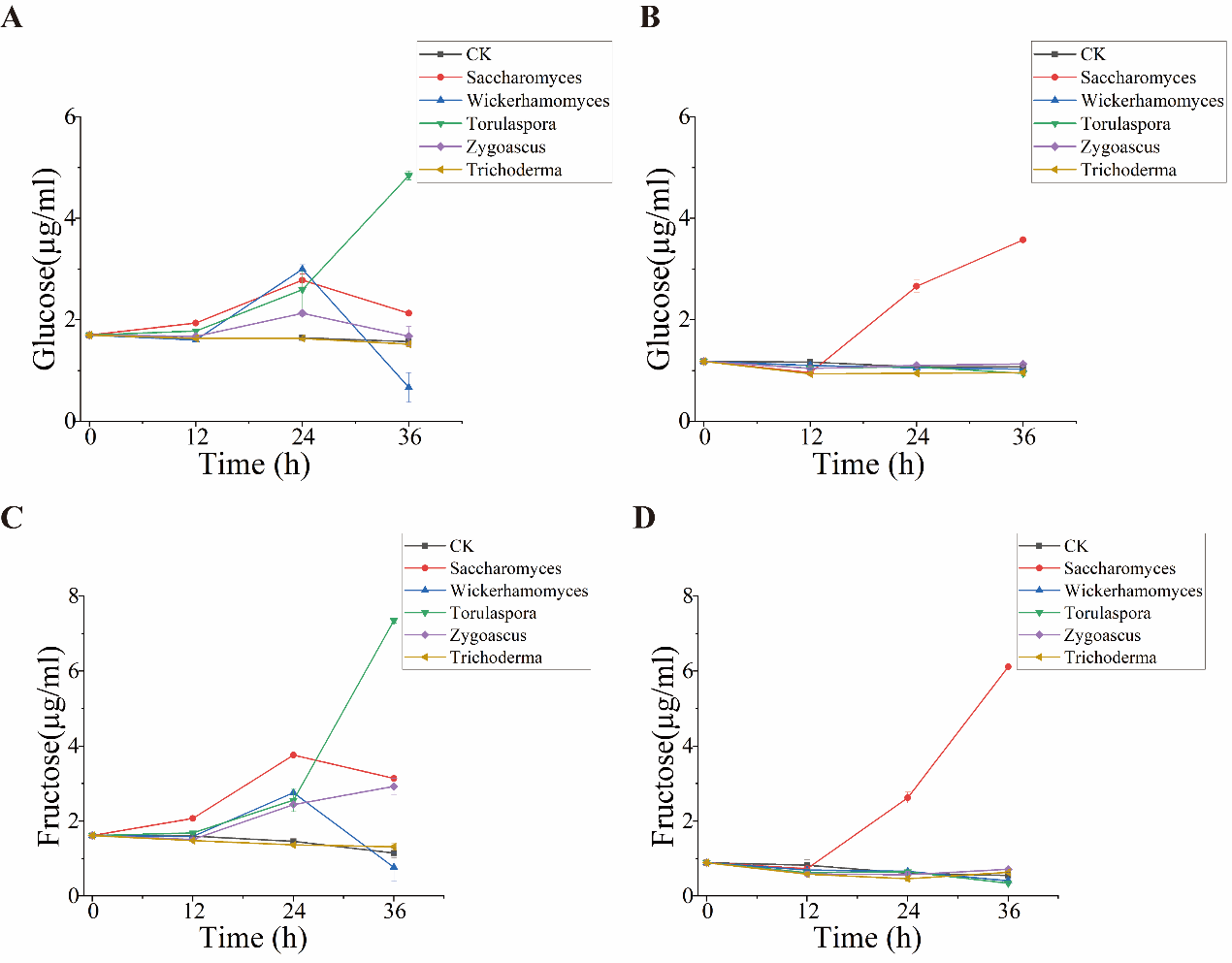


**Fig S2. The glucose and fructose profiles in sucrose medium of isolated strains cultured at 30℃and 40℃.**

(a) glucose at 30℃; (b) glucose at 40℃; (c) fructose at 30℃; (d) fructose at 40℃.

Data are presented as the mean ± standard deviation.


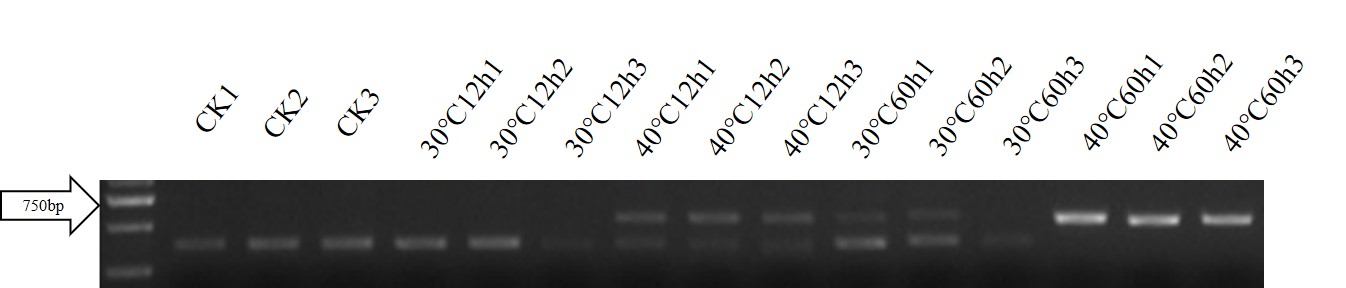


**Fig S3.** **PCR amplification for amplicon library construction using ITS21F/ITS22R primer pair.**

Lane 1: DNA marker; Lanes 2-4 (CK): before storage; Lanes 5-7 (30℃12h): stored 12h in 30℃; Lanes 8-10 (40℃12h): stored 12h in 40℃; Lanes 11-13 (30℃60h): stored 60h in 30℃; Lanes 14-16 (40℃60h): stored 60h in 40℃.
